# Antihyperglycemic and pancreatic tissue-preserving effects of *L. sativum* oil in streptozotocin-induced diabetic rats: exploratory analysis of PDX-1 and GCK

**DOI:** 10.3389/fendo.2026.1963673

**Published:** 2026-10-07

**Authors:** Fatimah A. Al-Saeed, Nadiyah M. Alabdallah

**Affiliations:** 1 Department of Biology, College of Science, King Khalid University, Abha, Saudi Arabia; 2 Department of Biology, College of Science, Imam Abdulrahman Bin Faisal University, Dammam, Saudi Arabia; 3 Basic & Applied Scientific Research Centre, Imam Abdulrahman Bin Faisal University, Dammam, Saudi Arabia

**Keywords:** GCK, glycemic control, *L. sativum*, oxidative stress, PDX-1, streptozotocin-induced diabetes

## Abstract

**Background:**

Diabetes mellitus constitutes a major global health burden. *L. sativum* has long been employed in traditional medicine for glycemic management, an effect attributed to its rich phytochemical constituents, including flavonoids, phenolic acids, and glucosinolates. This study investigated the ameliorative effects of *L. sativum* oil against streptozotocin-induced type 1 diabetes in rats, with particular emphasis on pancreatic and duodenal homeobox 1 (PDX-1) and glucokinase (GCK) gene expression.

**Methods:**

A total of 48 young male Wistar rats were randomly divided into four experimental groups (n = 12 per group): a control group (CG), a streptozotocin-induced type 1 diabetic group (SG; 60 mg/kg, single intraperitoneal injection), a diabetic group treated with *L. sativum* oil group (S+*LS*G; 250 mg/kg/day, oral gavage), and a healthy group receiving *L. sativum* oil alone (LSG; 250 mg/kg/day).

**Results:**

In this study, the SG exhibited significant hyperglycemia, hypoinsulinemia, and dyslipidemia compared to the CG (P < 0.01). Treatment of diabetic rats with *L. sativum* oil (S+LSG) markedly attenuated these metabolic abnormalities, lowering blood glucose to 146.33 mg/dL, raised insulin to 15.51 µIU/mL, and improving the lipid profile relative to the SG. Histological analysis indicated severe islet atrophy, extensive cytoplasmic vacuolation, and interstitial fibrosis in the SG group, while the S+LSG group displayed notable architectural improvement and preserved islet integrity. Analysis of serum oxidative stress markers revealed that the SG exhibited significantly reduced (P < 0.01) activities of glutathione (GPx), superoxide dismutase (SOD), and catalase (CAT) compared to the CG. Furthermore, treatment with *L. sativum* (S+LSG) significantly restored these enzyme activities to GPx, SOD, and CAT. In contrast, serum MDA levels were markedly elevated in the SG relative to the CG, and were partially reduced in the S+LSG. Gene expression analysis revealed significant downregulated in the PDX-1 (0.802-fold) and GCK (0.779-fold) in the SG compared to CG (P < 0.01). Treatment with *L. sativum* oil in the diabetic rats (S+LSG) resulted in partial restoration (PDX 1: 0.830-fold and GCK: 0.840-fold); however, these changes were not statistically significant relative to SG.

**Conclusion:**

*L. sativum* oil may effectively alleviate hyperglycemia, preserve pancreatic tissue architecture, and enhance antioxidant defense mechanisms in streptozotocin-induced diabetic rats.

## 1 Introduction

Diabetes mellitus (DM) is a chronic metabolic disorder of global concern, characterized by persistent hyperglycemia resulting from defects in insulin action, insulin secretion, and/or both (1). The progressive decline in pancreatic β-cell function and mass is a hallmark of DM, ultimately leading to impaired glucose-stimulated insulin secretion (2). Existing treatments, while valuable, may not always reverse this trend and may have side effects (3), underscoring the urgent need for novel, multi-targeted therapies. In this context, medicinal plants have emerged as a valuable source for bioactive components with pleiotropic mechanisms of action (4, 5). *L. sativum*, a member of the Brassicaceae family, has been traditionally used in the Middle East and North Africa for the management of DM and hypertension (6). The phytochemical profile of *L. sativum* includes flavonoids (e.g., catechins), phenolic acids (e.g., gallic, ellagic, and chlorogenic acid), and glucosinolates, which are known to exert potent antioxidant and anti-inflammatory activities (7). Experimental studies have demonstrated that *L. sativum* oil effectively reduces fasting blood glucose, improves insulin concentration, and ameliorates diabetes- associated dyslipidemia in animal models (8). Furthermore, *L. sativum* treatment has been shown to enhance the activities of endogenous antioxidant enzymes, including superoxide dismutase (SOD), glutathione peroxidase (GPx), and catalase (CAT), while suppressing lipid peroxidation, as indicated by reduced malondialdehyde (MDA) concentration (9, 10). Histological evidence also indicates that *L. sativum* can reverse pancreatic β-cell damage and restore islet architecture in DM, accompanied by notable improvements in diabetes-related dyslipidemia (11). Additionally, *L. sativum* treatment is linked to improved diabetic nephropathy symptoms, characterized by decreased blood urea, creatinine, and albumin excretion, while preserving renal histological structure (9). The single high-dose streptozotocin (STZ; 60 mg/kg body weight) model induces a type 1 diabetes-like phenotype, characterized by substantial pancreatic β-cell destruction and marked insulin deficiency, rather than the insulin resistance and progressive β-cell dysfunction that typify type 2 diabetes (12). At the molecular level, the inflammatory and oxidative milieu characteristic of streptozotocin-induced diabetes has been shown to downregulate the expression of two critical regulators of β-cell identity and function: pancreatic and duodenal homeobox 1 (PDX-1) and glucokinase (GCK) gene expression (1, 13). Glucokinase functions as the primary glucose sensor in pancreatic β-cell, catalyzing the rate-limiting phosphorylation of glucose and thereby governing glucose-stimulated insulin secretion (GSIS) (14). Similarly, PDX-1 is a master transcriptional factor that orchestrates pancreatic development, β-cell differentiation, and the maintenance of mature β-cell function by directly activating the insulin gene (15). Although the glycemic-regulating effects of *L. sativum* have been documented in several studies, further research is needed to determine whether these effects are mediated by its influence on genes involved in β-cell identity and function, such as PDX-1 and GCK, by its histological effects on pancreatic tissue, or by both mechanisms. Accordingly, this study tested the hypothesis that *L. sativum* oil directly modulates the expression of PDX-1 and GCK in pancreatic tissue, thereby regulating blood glucose levels and influencing pancreatic histology. Thus, this study offers a novel perspective by exploring the potential effects of *L. sativum* oil on glycemic control effects, lipid profiles, and oxidative stress markers, with a particular focus on the PDX-1 and GCK expression. The study aimed to: (1) evaluate the effect of *L. sativum* oil on fasting blood glucose, insulin concentrations, lipid profile and oxidative stress markers in streptozotocin (STZ)-induced diabetic rats. (2) assess histopathological change in pancreatic tissue; and (3) quantify the relative mRNA expression levels of PDX-1 and GCK.

## 2 Materials and methods

### 2.1 Animals grouping and management

Forty-eight young male Wistar rats (*Rattus norvegicus*) weighing 212.01 ± 5.25 g (mean ± SD) and aged 6 weeks, were included in the current study. The study protocol was reviewed and approved by the Research Ethics Committee at King Khalid University; Reference No. KKU-173-2025-31. Following a 14-day acclimatization period, the animals were randomly allocated to four experimental groups (n = 12 per group), using a computer-generated random number sequence. Allocation concealment was achieved by sequentially numbering opaque, sealed envelopes that contained the group assignment, which were opened only at the time of intervention assignment. A G*Power, one-way ANOVA, 4group, α = 0.05, power = 0.80, confirmed that 12 rats are adequate and consistent to sample size. Group I (Control group, CG): received distilled water at a volume equivalent to that used by the treated groups. Group II (Streptozotocin group, SG): received a single intraperitoneal (IP) injection of a freshly prepared streptozotocin (STZ) solution (60 mg/kg body weight) to induce diabetes. Group III (Streptozotocin + *L. sativum* Oil group, S+*LS*G): received STZ solution (60 mg/kg body weight; IP), and after confirmation of diabetes (48–72 h post STZ injection, as described in Section 2.2), rats received *L. sativum* oil at a dose of 250 mg/kg body weight daily, via oral gavage for 28 days. Group IV (*L. sativum* oil, *LS*G): received *L. sativum* oil at a dose of 250 mg/kg body weight daily, also via oral gavage for 28 days, without STZ administration. The 250 mg/kg dose of *L. sativum* was selected based on previous rodent studies demonstrating its safety and efficacy (9, 16). The Certificate of *L. sativum* oil is presented in **Table 1**. The animals were maintained under standard laboratory conditions in polypropylene cages (40×25×20 cm, max 5 rats/cage), at a temperature of 22 ± 2 °C, and under 12-hour light/dark cycles. The animals were housed in a ventilated system to ensure continuous air exchange and minimize contaminant buildup, with humidity levels ranging from 45 to 55%, with light intensity at cage level maintained at approximately 50–100 lux. Standard rodent pellet feed and tap water were provided ad libitum.

**Table 1:** Certificate of *L. sativum* oil*.

Item	Specification	Result	Test method
Appearance	Clear to pale yellow oily liquid	Conforms	Visual
Color	Pale yellow	Conforms	Visual/Colorimeter
Odor	Characteristic odor of Garden Cress oil	Conforms	Organoleptic
Specific Gravity	0.915 - 0.935 (at 20 °C)	0.925	USP <841> or equivalent
Refractive Index	1.465 - 1.485 (at 20 °C)	1.475	USP <831> or equivalent
Acidity	≤ 2.0 mg KOH/g	1.5	Titration
Peroxide Value	≤ 10 meq/kg	4.0	Iodometric
Loss on Drying	≤ 0.5%	0.2%	USP <731> or equivalent

*The certificate of analysis from Purita, a subsidiary of Tower CV of Hailing International E&T in Xi’an, Shaanxi Province, China, includes quality control results for *L. sativum* oil and presents physical and quality parameters as specified by the manufacturer.

### 2.2 Induction of the diabetes model

Streptozotocin (Zanosar^®^; CAS No. 18883-66-4; C_8_H_15_N_3_O_7_; molecular weight: 265.22 g/mol), purchased from Sigma-Aldrich (Catalog No. S0130, St. Louis, MO, USA), was dissolved in ice-cold 0.1 M sodium citrate buffer (pH 4.5) prior to use to ensure stability. The resultant solution was immediately sealed with aluminum foil to protect it from light, and was used within 15 minutes to prevent decomposition (17). After a 14-day acclimatization period, all animals were fasted overnight (12 h) with free access to water before injection, as recommended for STZ-induced diabetes protocols. Animals in groups II and III were injected with a single dose (60 mg/kg body weight; IP) of the freshly prepared STZ solution via the IP route using a 26-gauge needle and a 1 mL syringe; injection volume was calculated based on the individual weight of each animal. Animals in groups I and IV received an equivalent volume of 0.1 M sodium citrate buffer (18). To prevent mortality, animals were immediately administered 5% glucose solution orally post-injection. Diabetes was confirmed 48–72 h after STZ injection by measuring fasting blood levels in all rats. All STZ-injected animals (n = 24 animals) developed hyperglycemia (> 250 mg/dL) and were confirmed diabetic. No animals were excluded or mortality from this study, resulting in 12 animals per group for final analysis. Treatment with L. sativum oil began in Group III after diabetes was confirmed.

### 2.3 Biochemical analyses

Following the 4-week *L. sativum* oil treatment and STZ injection, blood samples (n = 48; one sample per animal) were collected from the retro-orbital plexus using a heparinized capillary tube under deep anesthesia induced by an intraperitoneal injection of ketamine (50 mg/kg) and xylazine (5 mg/kg). Immediately after blood collection, euthanasia was performed by cervical dislocation. Serum was separated by centrifugation (3,000 × g for 20 minutes) and stored at −20 °C until analysis. Fasting blood levels (12 hours) in serum glucose concentration was measured using the glucose oxidase peroxidase aminophenazone (GOD-PAP) method [Vitro Scient; (19)], and insulin concentration was assessed using an enzyme-linked immunosorbent assay (ELISA) kit with a standard sensitivity of 0.135 μIU/ml (20). Serum antioxidant enzyme activities: glutathione peroxidase (GPx), superoxide dismutase (SOD), and catalase (CAT) were measured using colorimetric kinetic assay kit [Bio-Diagnostic c. (21, 22)]. Additionally, malondialdehyde (MDA) concentration, a marker of lipid peroxidation, was determined using a thiobarbituric acid reactive substances (TBARS) colorimetric assay kit. Serum total protein and albumin concentrations (g/dL) were determined using assay kits [SPINREACT, S.A., Ctra. Santa Coloma, Girona, Spain (23)]. Commercial colorimetric kits (spinreact, Girina, Spain) according to the manufacturer instructions, serum lactate dehydrogenase (LDH), and high-density lipoprotein (HDL) were performed, while total cholesterol (TC; mg/dL) was measured by the cholesterol oxidase peroxidase amino phenazone (CHOD-PAP) method. Aspartate transaminase (AST), alanine transaminase (ALT) were performed by using Biomed Kinetic kits (Cairo, Egypt) (24). Serum urea, creatinine, uric acid (mg/dL) were measured by kinetic colorimetric methods as described by Tietz (25) using commercially available kits from Biomed Diagnostics (EGY-MDSS for Lab Technology, Cairo, Egypt).

### 2.4 Histopathological examination

After blood samples were collected, pancreatic tissues were excised from all animals in each group. Each tissue was divided into two portions: The portion assigned for histology (n = 12 per group) was carefully washed with sterile saline (0.9% NaCl) and fixed in 10% neutral buffered formalin (NBF) for histopathological examination. The other portion was immediately stored at -80 °C for quantitative real-time PCR (qPCR; n = 12 per group) analysis. The samples were trimmed into small pieces (5 mm thick) and immediately fixed for 48 hours at room temperature to ensure optimal preservation of tissue architecture and prevent autolytic degradation. Subsequently, the fixed pancreas specimens were dehydrated through a graded series of ethanol concentrations (70%, 80%, 90%, 95%, and 100%), cleared in xylene to remove the dehydrating agent, and infiltrated with molten paraffin wax in a 58-60 °C oven. Using a microtome, serial sections (4–5 μm thick) were cut, mounted on positively charged glass slides, and routinely stained with hematoxylin and eosin (H&E) to assess overall histological and pathological changes. The stained pancreas sections were examined under a compound light microscope (Luxeo 4D model, Labomed, El Segundo, CA, USA).

High-resolution digital images were captured from five randomly selected non-overlapping microscopic fields per slide at 200× and 400× magnifications, using a calibrated 100 μm scale bar for standardization. For semiquantitative histopathological scoring, three non-overlappingsection per animal were analyzed. From each section, five randomly selected non-overlapping fields were examined at 400× magnifications. Each of the four parameters (islet atrophy, cytoplasmic vacuolation, inflammatory cell infiltration, and interstitial fibrosis) were scored on a 0–4 scale as follows: 0, normal; 1, minimal; 2, mild; 3, moderate; and 4, severe. The scores from the fields were averaged to yield a single mean score per animal for each parameter. Therefore, each data point represents the mean score of one animal (n = 12 animal per group). To ensure objectivity and minimize observer bias, all histopathological assessments were performed by an experienced pathologist who was blinded to the treatment group allocation.

### 2.5 Quantitative real-time PCR (qPCR) analysis of GCK and PDX-1 gene expression

#### 2.5.1 Total RNA extraction and quality assessment

Total RNA extraction from frozen pancreatic tissue (50–100 mg) was performed using TRIzol™ Reagent (Cat. No. 15596026, Life Technologies, Carlsbad, CA, USA), according to the manufacturer’s protocol. The tissue was homogenized in 1 mL of TRIzol™ at 6,000 rpm for 30 seconds, with a 30-second cooling interval. After incubating the homogenate at room temperature for 5 minutes, chloroform was added, and the mixture was vortexed and incubated again. Phase separation occurred through centrifugation, resulting in three layers: a lower phenol-chloroform phase, a white interphase, and a colorless upper aqueous phase containing RNA. The RNA was then pelleted through centrifugation, washed with 75% ethanol, and air-dried. Finally, the pellet was resuspended in RNase-free water and heated at 55–60 °C to promote solubilization. RNA concentration and purity were measured using a NanoDrop™ 2000 spectrophotometer at 260 nm and 280 nm. Integrity was evaluated via formaldehyde-agarose gel electrophoresis to visualize 28S and 18S ribosomal RNA bands, maintaining an intensity ratio of approximately 2:1. Acceptable RNA samples had A260/A280 ratios between 1.8 and 2.1, A260/A230 ratios above 1.8, and intact ribosomal RNA bands. Extracted RNA was aliquoted and stored at −80 °C to avoid degradation.

#### 2.5.2 Genomic DNA elimination and reverse transcription

Prior to reverse transcription, genomic DNA (gDNA) was eliminated using the gDNA Wipeout Buffer from the QuantiTect^®^ Reverse Transcription Kit. The reaction, conducted on ice, included 2 µL of the gDNA Wipeout Buffer, 1 µg of total RNA, and RNase-free water, to a final volume of 14 µL. After gentle vortexing and brief centrifugation, the mixture was incubated at 42°C for 2 minutes before being cooled on ice. First-strand complementary DNA (cDNA) was synthesized by adding 6 µL of reverse transcription master mix to a 14 µL genomic DNA elimination reaction, totaling 20 µL. The mix included 4 µL of 5× Quantiscript^®^ RT Buffer, 1 µL of Quantiscript^®^ Reverse Transcriptase, and 1 µL of RT Primer Mix. The reaction was mixed, centrifuged, and incubated at 42 °C for 15 minutes, then heat-inactivated at 95 °C for 3 minutes. The cDNA was diluted 1:10 with RNase-free water and stored at −20 °C in aliquots.

#### 2.5.3 Primer and quantitative real-time PCR amplification

Gene-specific primers for glucokinase (GCK), pancreatic and duodenal homeobox 1 (PDX-1), and the housekeeping gene β-actin were designed using the National Center for Biotechnology Information (NCBI) Primer-BLAST tool (https://www.ncbi.nlm.nih.gov/tools/primer-blast/). Selection criteria included an amplicon length of 80–200 base pairs, a melting temperature of 58–62 °C, GC content of 40–60%, and the absence of self-complementarity and primer-dimer formation. Specificity was confirmed through BLAST analysis against the *Rattus norvegicus* reference genome. Detailed primer sequences and references are provided in **Table 2**.

**Table 2:** Primer sequences used for quantitative real-time PCR analysis of GCK, PDX-1, and β-actin gene expression.

Genes	Primer sequence (5’->3’)	Primer Tm	Primer GC%	Amplicon size (bp)	References
GCK	Fw: AGGTGATGAGCCGGATGCAG	62.32	60.00	159	(26)
	Rev: CCCTCTCCCACTTTGACCAG	59.67	60.00		
PDX-1	For: CTCGCTGGGAACGCTGGAACA	65.22	61.90	225	(27)
	Rev: GCTTTGGTGGATTTCATCCACGG	62.53	52.17		
β-actin	Fw: GTGACATCCACACCCAGAGG	60.14	52.38	141	(28)
	Rev: ACAGGATGTAAAACTGCC	60.12	57.14		

Quantitative real-time PCR was conducted using a Rotor-Gene Q analyzer and Maxima™ SYBR Green/Fluorescein qPCR Master Mix. Each 25 µL reaction consisted of the master mix, optimized primers, cDNA template, and nuclease-free water. The protocol included an initial denaturation at 95 °C for 10 minutes, followed by 45 amplification cycles of denaturation at 95 °C for 10 seconds, annealing at 60 °C for 15 seconds, and extension at 72 °C for 15 seconds. Fluorescence was recorded after each extension step. Melting curve analysis was performed from 72 °C to 95 °C with a transition rate of °C per second. No-template controls were included in each run. PCR efficiency was 98.2% for GCK, 101.5% for PDX-1, and 96.8% for β-actin, with correlation coefficient (R²) 0.992, 0.989, and 0.995, respectively. The β-actin (housekeeping gene) was selected as the internal reference based on its consistent amplification among groups, and raw CT values were comparable among experimental group, supporting its use for normalization of PDX-1 and GCK expressions.

#### 2.5.4 Data analysis and normalization

The cycle threshold (Ct) value, which indicates the point at which fluorescence exceeds a set threshold, was determined using Rotor-Gene Q Series Software with consistent settings for each gene. Relative quantification of gene expression utilized the comparative cycle threshold method [2^(−ΔΔCt)] as per Livak and Schmittgen (29), Ct values of the target genes (GCK and PDX-1) were normalized against the housekeeping gene β-actin to yield ΔCt values. Experimental groups’ ΔCt values were then compared to the control group’s mean to compute ΔΔCt values. Each sample underwent triplicate analysis, using the mean Ct value for further analysis, relative expression was calculated as 2^(−ΔΔCt), with the control assigned a value of 1.0, indicating baseline expression. No-template controls (NTC) confirmed the absence of contamination and nonspecific products, as all showed no amplification. Given the 45-cycle protocol, true no-amplification was defined as CP undetermined. Any NTC with a Cq > 40 locked a specific melting peak and was not considered true amplification.

### 2.6 Statistical analysis

Statistical analysis was performed using SPSS for Windows (version 25.0, IBM Corp., Armonk, NY, USA). The normality of distribution was assessed using Shapiro–Wilk test. Data were analyzed using one-way analysis of variance (ANOVA) followed by Tukey’s HSD *post-hoc* test pairwise comparisons among the experimental groups. The statistical model was structured according to the generalized linear model: Y_ij_ = μ + T_i_ + Eij, where Y_ij_ represented the experimental observation, μ is the overall mean, T_i_ denotes the treatment effect (i = 1, 2, 3, and 4; corresponding to CG, SG, S+*LS*G, and *LS*G, respectively), and E_ij_ is the random experimental error. Semi-quantitative histopathological scoring (0-4) were analyzed using the Kuskal-wallis test followed by Dunn’s *post-hoc* test, and were presented as median (inter quartile range; IQR). A probability level of P < 0.01 was considered statistically significance, with data presented as mean ± standard error of mean (SEM) for each experimental group (n = 12). Exact P values are provided when available; P-values greater than 0.0001 were reported to < 0.0001, and P-values less than 0.0001 and greater than 0.001 were reported as < 0.001.

## 3 Results

### 3.1 Glucose, insulin, and lipid profile

Fasting blood glucose levels (mg/dL; **Table 3**) were significantly higher in the streptozotocin-induced diabetic group (SG; 349.35) compared to the control group (CG; 136.82). However, treating diabetic rats with *L. sativum* oil decreased fasting blood glucose to 146.33 mg/dL, which was significantly lower than SG (P<0.01) and not significantly different from CG (P>0.05), indicating normalization of glycemia. Treatment with *L. sativum* oil in the LSG group exhibited normoglycemia (129.01), with no significant difference to the CG (P > 0.05). Serum insulin concentrations (µIU/mL) were significantly lower in the SG (4.85) compared to the CG (21.74). Treatment with *L. sativum* oil in the S+LSG increased insulin levels to 15.51, though this was still significantly lower than CG levels. The LSG maintained insulin levels (21.18) near to the CG (P > 0.05). Regarding lipid profile, the SG showed significant dyslipidemia (P < 0.01), characterized by elevated levels of total cholesterol (TC), triglyceride (Tg), and low-density lipoprotein (LDL) alongside a significant reduction in high-density lipoprotein (HDL) compared to both the CG and *LS*G. Additionally, treatment with *L. sativum* oil to diabetic rats in the S+LSG demonstrated a significant (P < 0.01) improvement in the TC, Tg, LDL, and HDL compared to the diabetic group (SG). The LSG group displayed blood glucose and insulin concentrations comparable to those of the CG (P > 0.05; **Table 3**). However, HDL was significantly lower in the LSG than in the CG, whereas LDL did not differ significantly between LSG and CG.

**Table 3:** Effect of oral administration of *L. sativum* oil (250 mg/kg body weight/day for 28 days) on fasting blood glucose, serum insulin, and lipid profile in streptozotocin-induced diabetic Wistar rats.

Parameter (Unit)	CG	SG	S+LSG	LSG	P-value
Glucose (mg/dL)	136.82 ± 1.66 ^b^	349.35 ± 9.80 ^a^	146.33 ± 2.26 ^b^	129.01 ± 1.99 ^b^	<0.0001
Insulin (µIU/mL)	21.74 ± 0.73 ^a^	4.85 ± 0.29 ^c^	15.51 ± 0.48 ^b^	21.18 ± 0.40 ^a^	<0.0001
Total Cholesterol (TC)	73.35 ± 1.34 ^c^	118.83 ± 2.17 ^a^	83.50 ± 1.57 ^b^	68.75 ± 1.12 ^c^	<0.0001
Triglyceride (Tg)	111.67 ± 1.58 ^c^	251.50 ± 2.91 ^a^	183.33 ± 2.51 ^b^	102.08 ± 4.22 ^c^	<0.0001
Low-density lipoprotein (LDL)	54.88 ± 0.94 ^c^	116.91 ± 7.81 ^a^	88.75 ± 2.15 ^b^	47.58 ± 0.35 ^c^	<0.0001
High-density lipoprotein (HDL)	39.09 ± 1.03 ^a^	22.58 ± 0.68 ^c^	31.67 ± 1.61 ^b^	33.17 ± 1.05 ^b^	<0.0001

Values are presented as mean ± SEM (n = 12 per group). CG, Control group; SG, Streptozotocin-induced diabetic group; S+LSG, Diabetic group treated with *L. sativum* oil; LSG, Healthy group treated with *L. sativum* oil only. Statistical analysis: One-way ANOVA followed by Tukey’s *post-hoc* test. Different superscript letters (^a,b,c^) within the same row indicate significant differences at P < 0.01. P-values greater than 0.0001 were reported to < 0.0001.

### 3.2 Histological examination

Hematoxylin and eosin (H&E)-stained sections from the CG exhibited normal pancreatic architecture (**Figure 1A**). The islets of Langerhans were well preserved, retained their typical morphology, and displayed no degeneration, necrosis, inflammation, or fibrosis changes, indicating no pathological alterations under normal conditions. In contrast, streptozotocin-induced diabetes in the SG caused several pancreatic histopathological alterations, including atrophic islets of Langerhans, marked cellular disintegration, and extensive cytoplasmic vacuolation. The endocrine-exocrine tissues boundary was disrupted, with observed interstitial lymphocytic infiltration and interstitial fibrosis. Adjacent exocrine tissue also showed degenerative changes (**Figure 1B**). In the S+LSG, diabetic rats treated with *L. sativum* oil showed histopathological improvement in the pancreatic section (**Figure 1C**). The islets of Langerhans exhibited a relatively well-organized structure, with diminished cytoplasmic vacuolation and enhanced cellular integrity; however, they did not completely match the level observed in CG. Only minor degenerative changes were noted in the adjacent exocrine tissue. These findings indicate preservation of pancreatic architecture rather than regeneration. Furthermore, in the LSG, The *L. sativum* oil-treated group exhibited histopathological normal pancreatic tissue. The islets of Langerhans showed typical morphology and preserved cellular density, with no signs of degeneration or inflammation. The adjacent exocrine tissue also appeared normal and retained its acinar structure and lobular organization (**Figure 1D**).

**Figure 1:**
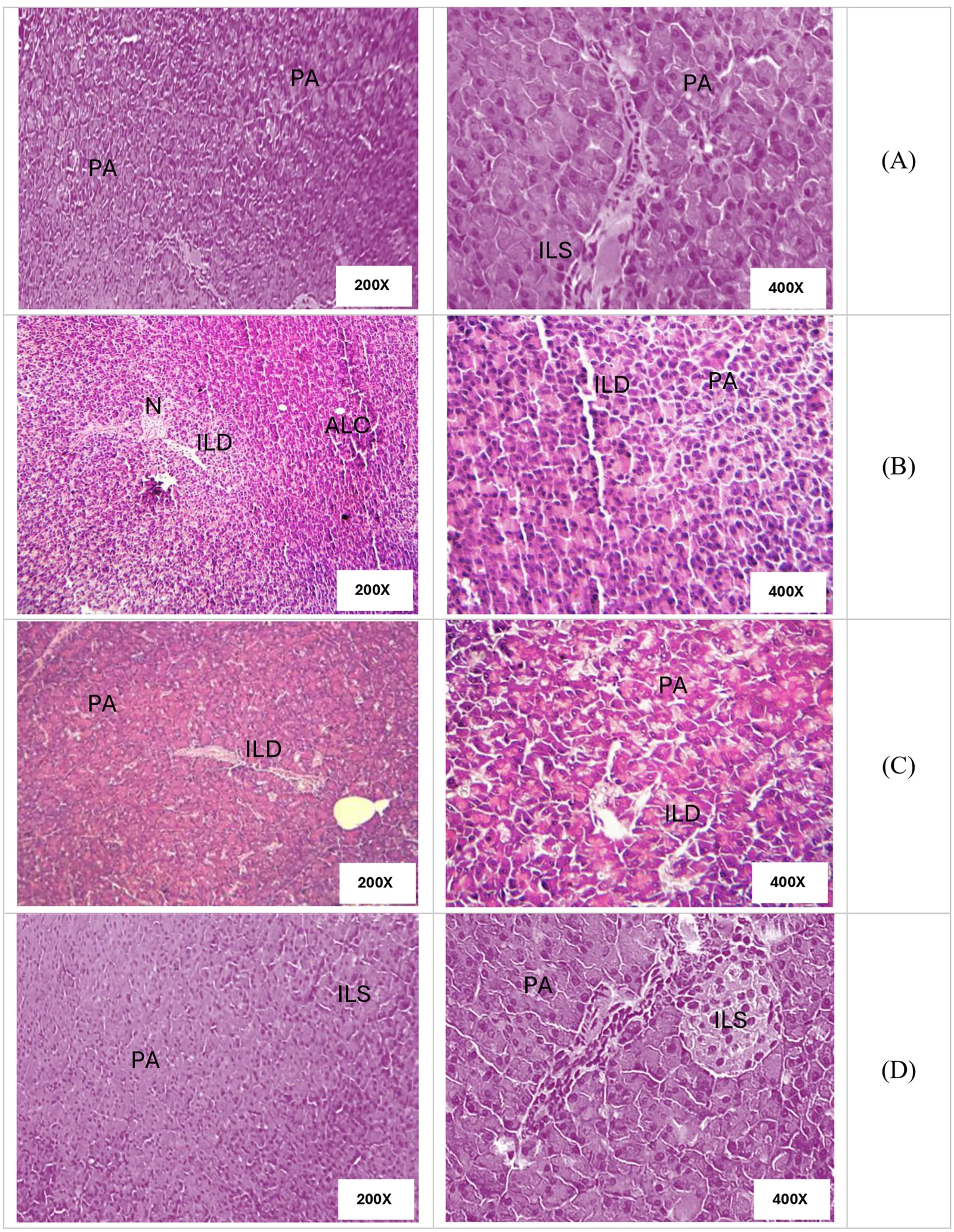
Illustrates a comparative study of pancreatic sections stained with H&E. **(A)** shows the control group with normal architecture. **(B)** depicts the diabetic group suffering from STZ-induced injury, evident through atrophic islets, cytoplasmic vacuolation, inflammatory aggregates, necrosis, and interstitial fibrosis. **(C)** presents improvements in the diabetic group treated with *L. sativum* oil, displaying better islet organization and reduced vacuolation. **(D)** features the *L. sativum* oil-only group, which exhibits normal pancreatic tissue and typical islet morphology. PA, pancreatic acini; ILD, intralobular duct; ILS, islets of Langerhans; AIC, aggregates of inflammatory cells; N, necrosis. Magnification: ×200 and ×400.

Semi-quantitative histopathological scoring of pancreatic tissues (**Table 4**) revealed that streptozotocin-induced diabetic rats (SG) exhibited significantly higher score (P < 0.01) for islet atrophy, cytoplasmic vacuolation, inflammatory infiltration, and interstitial fibrosis compared to the control group (CG) and *L. sativum* group (LSG). Oral administration of *L. sativum* oil to diabetic rats (S+SLG) significantly reduced these median (IQR) scores relative to the SG (P < 0.01); however, they remained significantly higher than those in the CG and LSG (P < 0.01).

**Table 4:** Semi-quantitative histopathological scoring of pancreatic tissues after oral administration of *L. sativum* oil (250 mg/kg body weight/day for 28 days), in streptozotocin-induced diabetic Wistar rats.

Parameter	CG	SG	S+LSG	LSG	P-value
Islet atrophy	0 (0-0) ^c^	3 (2.5-4) ^a^	1 (1-1.5) ^b^	0 (0-0) ^c^	<0.0001
Cytoplasmic vacuolation	0 (0-0) ^c^	3 (3-3.5) ^a^	2 (1-2) ^b^	0 (0-0) ^c^	<0.0001
Inflammatory infiltration	0 (0-0) ^c^	3 (2-4) ^a^	1 (1-2) ^b^	0 (0-0) ^c^	<0.0001
Interstitial fibrosis	0 (0-0) ^c^	2.5 (2-3) ^a^	1.5 (1-2.5) ^b^	0 (0-0) ^c^	<0.0001

Values are presented as mean (IQR); n = 12 per group. Each animal provided a single averaged histopathological score, calculated from 15 fields by analyzing three non-overlapping sections with five randomly selected fields per section, using the animal as the unit of analysis. CG, Control group; SG, Streptozotocin-induced diabetic group; S+LSG, Diabetic group treated with L. sativum oil; LSG, Healthygroup treated with *L. sativum* oil only. Statistical analysis: Kruskal-Wallis test followed by Dunn’s *post-hoc* test. Different superscript letters (^a,b,c^) within the same row indicate significant differences at P < 0.01. P-values greater than 0.0001 were reported to < 0.0001.

### 3.3 Oxidative stress parameter

The serum activities of the oxidative stress glutathione peroxidase (GPx), superoxide dismutase (SOD), and catalase (CAT) were significantly lower in the streptozotocin-induced diabetic group (SG) compared to the control group (CG), with respective values of 13.07, 15.74, and 25.75 for SG versus 34.86, 24.77, and 89.36 for CG, respectively (P < 0.01; **Table 5**). In the diabetic rats, oral administration of *L. sativum* oil significantly improved serum antioxidant enzyme levels, producing GPx, SOD, and CAT values of 31.33, 21.57 and 51.65, respectively (P < 0.01). Furthermore, serum malondialdehyde (MDA) concentrations exhibited significant lipid peroxidation in the SG, with levels at 2.27 compared to 0.95 in the CG (P < 0.01). Notably, treatment with *L. sativum* oil in the S+LSG group markedly reduced the MDA level to 1.68 relative to 2.27 in the SG (P < 0.01). The lowest serum MDA concentration (0.93) was observed in the *LS*G group, which was not significantly different from that of the CG (P > 0.05).

**Table 5:** Effect of oral administration of *L. sativum* oil (250 mg/kg body weight/day for 28 days) on oxidative markers in streptozotocin-induced diabetic Wistar rats.

Parameter	CG	SG	S+LSG	LSG	P-value
Oxidative Stress					
Glutathione Peroxidase (GPx; U/mL)	34.86 ± 0.41 ^a^	13.07 ± 0.22 ^c^	31.33 ± 0.64 ^b^	33.27 ± 0.59 ^a^	<0.0001
Superoxide Dismutase (SOD; U/mL)	24.77 ± 0.66 ^a^	15.74 ± 0.39 ^c^	21.57 ± 0.24 ^b^	24.74 ± 0.49 ^a^	<0.0001
Catalase (CAT; U/mL)	89.36 ± 1.78 ^a^	25.75 ± 0.29 ^c^	51.65 ± 1.60 ^b^	91.61 ± 1.10 ^a^	<0.0001
Lipid peroxidation					
Malondialdehyde (MDA; nmol/mL)	0.95 ± 0.01 ^c^	2.27 ± 0.12 ^a^	1.68 ± 0.06 ^b^	0.93 ± 0.01 ^c^	<0.0001

Values are presented as mean ± SEM (n = 12 per group). CG, Control group; SG, Streptozotocin-induced diabetic group; S+LSG, Diabetic group treated with *L. sativum* oil; LSG, Healthy group treated with *L. sativum* oil only. Statistical analysis: One-way ANOVA followed by Tukey’s *post-hoc* test. Different superscript letters (^a,b,c^) within the same row indicate significant differences at P < 0.01. P-values greater than 0.0001 were reported to < 0.0001.

### 3.4 Kidney and liver function

Serum total protein and albumin (g/dL) concentrations were significantly lower (P < 0.01; **Table 6**) in the streptozotocin-induced diabetes group (SG) compared to the control group (CG). Furthermore, the SG exhibited significantly elevated levels of the alanine aminotransferase (ALT) and aspartate aminotransferase (AST) compared to the CG (P < 0.01), with respective values for ALT/AST (U/L) of 96.36/189.40 in SG and 63.15/131.26 in CG. In contrast, treatment with *L. sativum* oil in diabetic rats (S+*LS*G) showed significantly improved serum ALT and AST activities (80.21 and 162.47 U/L, respectively) compared to the SG, although both values remained higher than those in the CG and *L. sativum* oil groups (*LS*G). Serum levels of kidney function markers, including urea, creatinine, and uric acid concentrations (mg/dL) were significantly elevated in the SG compared to the CG and *LS*G (P < 0.01). In the S+*LS*G exhibited significantly improved (P < 0.01) urea, creatinine, and uric acid (63.67, 0.89 and 3.19, respectively) compared to the SG (81.93, 1.59 and 3.66, respectively), although both values remained higher than those in the CG and *LS*G.

**Table 6:** Effect of *L. sativum* oil (250 mg/kg body weight/day for 28 days, orally) on liver and kidney function in streptozotocin-induced diabetic Wistar rats.

Parameter (unit)	CG	SG	S+LSG	LSG	P-value
Liver Functions					
Total Protein (g/dL)	6.96 ± 0.14 ^a^	3.97 ± 0.07 ^c^	5.84 ± 0.21 ^b^	6.77 ± 0.07 ^a^	<0.0001
Albumin (g/dL)	3.48 ± 0.06 ^a^	2.49 ± 0.09 ^b^	2.84 ± 0.11 ^b^	3.31 ± 0.08 ^a^	<0.0001
alanine aminotransferase (ALT; U/L)	63.15 ± 0.90 ^c^	96.36 ± 0.90 ^a^	80.21 ± 1.18 ^b^	60.13 ± 0.49 ^c^	<0.0001
aspartate aminotransferase (AST; U/L)	131.26 ± 0.88 ^c^	189.40 ± 1.45 ^a^	162.47 ± 2.60 ^b^	133.19 ± 0.90 ^c^	<0.0001
Kidney Functions					
Urea (mg/dl)	46.58 ± 1.58 ^c^	81.93 ± 1.52 ^a^	63.67 ± 1.69 ^b^	43.82 ± 0.92 ^c^	<0.0001
Creatinine (mg/dl)	0.67 ± 0.01 ^c^	1.59 ± 0.04 ^a^	0.89 ± 0.01 ^b^	0.56 ± 0.01 ^c^	<0.0001
Uric Acid (mg/dl)	2.50 ± 0.08 ^c^	3.66 ± 0.10 ^a^	3.19 ± 0.05 ^b^	2.23 ± 0.06 ^c^	<0.0001

Values are presented as mean ± SEM (n = 12 per group). CG, Control group; SG, Streptozotocin-induced diabetic group; S+LSG, Diabetic group treated with *L. sativum* oil; LSG, Healthy group treated with *L. sativum* oil only. Statistical analysis: One-way ANOVA followed by Tukey’s *post-hoc* test. Different superscript letters (^a,b,c^) within the same row indicate significant differences at P < 0.01. P-values greater than 0.0001 were reported to < 0.0001.

### 3.5 GCK and PDX-1 gene expression

Glucokinase (GCK) gene expression was significantly downregulated (P < 0.01) in the streptozotocin-induced diabetic group (SG) compared to the control group (CG), exhibited a 22.1% reduction, with fold-change values of 0.779 and 1.00 (**Table 7**). In contrast, administration of *L. sativum* oil to diabetic rats (S+*LS*G) partially improve GCK expression to 0.840-fold (a 16.0% reduction), yet this improvement did not reach statistical significance relative to the SG (P > 0.05), and both diabetic groups remained markedly lower than the CG and *LS*G. Notably, the *LS*G exhibited only a marginal 9.9% decrease (0.901-fold), which was not statistically different from the CG (P > 0.05), confirming that the oil had no significant effect on GCK expression in healthy animals. Furthermore, PDX-1 expression was significantly downregulated in the diabetic group (SG) relative to the control group (CG), showing a 19.8% decrease (0.802 vs. 1.00, P < 0.01). In diabetic rats receiving *L. sativum* oil (S+LSG), expression partially recovered to 0.830-fold (a 17.0% reduction from CG), representing an absolute increase of 0.028 (i.e., 2.8% of the control value), or a relative improvement of 3.49% over the SG (0.802); however, this change was not statistically significant (P > 0.05). Meanwhile, healthy rats treated with the oil alone (LSG) exhibited a negligible decline (0.912-fold), with no significant difference from the CG (P > 0.05).

**Table 7:** Effect of *L. sativum* oil (250 mg/kg body weight/day for 28 days, orally) on GCK and PDX-1 gene expression in streptozotocin-induced diabetic Wistar rats.

Parameter (fold-change values)	CG	SG	S+LSG	LSG	P-value
PDX-1	1.00 ± 0.00 ^a^	0.802 ± 0.01 ^b^	0.830 ± 0.01 ^b^	0.912 ± 0.01 ^a^	<0.001
Glucokinase (GCK)	1.00 ± 0.00 ^a^	0.779 ± 0.06 ^b^	0.840 ± 0.05 ^b^	0.901 ± 0.02 ^a^	<0.001

Values are presented as mean ± SEM (n = 12 per group). CG, Control group; SG, Streptozotocin-induced diabetic group; S+LSG, Diabetic group treated with *L. sativum* oil; LSG, Healthy group treated with *L. sativum* oil only. Statistical analysis: One-way ANOVA followed by Tukey’s *post-hoc* test. Different superscript letters (^a,b,c^) within the same row indicate significant differences at P < 0.01. P-values less than 0.0001 and greater than 0.001 were reported as < 0.001.

## 4 Discussion

This study investigated the effect of *L. sativum* oil on blood glucose, insulin concentration, preservation of pancreatic tissue architecture, and antioxidant defense in streptozotocin-induced diabetic rats. It suggests that the observed benefits may be associated with of oxidative stress and possible tissue-preserving effects, rather than with significant upregulation of PDX-1 or GCK gene expression.

### 4.1 Glycemic control

The presented study reported that the SG exhibited significant hyperglycaemia, severe hypoinsulinaemia, and marked dyslipidaemia relative to the CG. These results align with the known diabetogenic effect of streptozotocin, which damages pancreatic beta-cells, impairs insulin secretion, and causes major metabolic disturbances (30). Furthermore, treatment with *L. sativum* oil in diabetic rats (S+LSG) regulated blood glucose, insulin concentrations, and marked dyslipidaemia compared to the SG (9). Additionally, the lipid-lowering effects may occur via increased hepatic low-density lipoprotein receptors and decreased activity of lipogenic enzymes such as 3-hydroxy-3methylglutryl-coenzyme A (HMG-CoA) reductase and fatty acid synthetase (31). Treatment with *L. sativum* (LSG) effectively maintained normal blood glucose and insulin levels, but it resulted in significantly lower HDL-cholesterol and creatinine levels compared to the control group. This indicates that while LSG does not exhibit overt toxicity, its safety profile may require further investigation to understand the significance of these metabolic effects.

### 4.2 Histopathological of the pancreatic tissue

Histological analysis of pancreatic tissues revealed significant damage, including atrophic islets of Langerhans, cytoplasmic vacuolation, lymphocytic infiltration, and interstitial fibrosis. These changes are attributed to the cytotoxic effects of streptozotocin, which induces β-cell necrosis through DNA damage and oxidative stress, leading to insulin insufficiency (32). Interstitial fibrosis signifies chronic pancreatic damage and remodeling associated with advanced diabetes mellitus in rodent models (33). However, there were significant histopathological improvements in the S+LSG group, evidenced by organized islets, reduced vacuolation, improved cellular integrity, and minimal degenerative changes in exocrine tissue compared to the SG. These observations are consistent with a possible tissue-preserving effect and the preservation of pancreatic tissue structure, rather than serving as conclusive evidence of beta-cell (β-cell) regeneration (34). *L. sativum* can scavenge reactive oxygen species (ROS), reduce lipid peroxidation, and mitigate the inflammatory response induced by streptozotocin through its antioxidant and anti-inflammatory properties (16). Furthermore, the treatment with *L. sativum* alone (LSG) displayed normal pancreatic architecture, indicating no toxicity.

### 4.3 Oxidative stress

The SG exhibited a significant decline in the serum activities of key endogenous antioxidant enzymes, including GPx, SOD, and CAT, alongside a marked elevation in serum MDA levels, indicative of enhanced lipid peroxidation and oxidative stress. These reflects the established role of streptozotocin-diabetes in generating ROS and depleting the cellular antioxidant defiance system (35). However, Treatment with *L. sativum* oil enhanced antioxidant enzymes (GPx, SOD, CAT) and reduced serum MDA levels in the S+LSG group compared to the SG. This improvement may be attributed to the high content of phenolic compounds and flavonoids in *L. sativum*, as hypothesized by Alsulaim et al. (9); these compounds scavenge free radicals and may activate the Nrf2 pathway, thereby enhancing endogenous antioxidant defense mechanisms. According to Dinić et al., maintaining redox balance is crucial for pancreatic β-cell function, as oxidative stress can cause dysfunction in diabetes (36).

### 4.4 Gene expression

At the molecular level, the streptozotocin-induced (SG) diabetes significantly downregulation of PDX-1 and GCK expression compared to the CG. However, in diabetic rats treated with *L. sativum* oil (S+LSG), both transcripts showed partial recovery in PDX-1 and GCK; however, these changes did not reach significance relative to the untreated diabetic group (SG). Several explanations may account for the dissociation observed. First, PDX-1 and GCK are regulated post-transcriptionally and post-translationally through mechanisms such as phosphorylation, acetylation, protein stability, and interaction with glucokinase regulatory protein (GKRP), indicating that mRNA levels may not accurately represent functional protein activity (15, 37). Second, *L. sativum* oil may protect surviving β-cells from oxidative damage and apoptosis, thereby preserving insulin secretory capacity without reactivating β-cell identity genes (38). Third, decreasing blood glucose and enhancing lipid profiles may lessen glucotoxicity and lipotoxicity, thereby indirectly benefiting β-cell function (1, 35). The LSG group showed similar PDX-1 and GCK expression levels to the CG, indicating that *L. sativum* oil does not significantly affect basal transcription in healthy pancreatic tissue.

### 4.5 Study limitations

One limitation of the presented study is the absence of a positive pharmacological control group, which precludes direct comparisons of the observed antihyperglycemic and pancreatic tissue-preserving effects with those of established therapies. This study explored the effects of *L. sativum* oil on streptozotocin-induced type 1 diabetes in rats, focusing on PDX-1 and GCK gene expression. The goal was to understand the intervention’s impact on pancreatic histology, oxidative stress markers, and β-cell gene expression, rather than to compare its effectiveness to standard treatments. Moreover, co-administration of a reference pharmacological agent could itself modulate glycemic control, oxidative stress, and β-cell function, thereby confounding the mechanistic endpoints assessed. Therefore, future studies should incorporate an appropriate positive control to enable valid comparisons and to evaluate the efficacy and potential clinical relevance of the intervention. Furthermore, this study is limited by its reliance on manufacturer-provided physical and quality parameters and by the absence of detailed chemical characterization of the *L. sativum* oil. Consequently, the attributed of the observed biological effects to specific phytochemical classes is based on previous literature. Future studies should include comprehensive chemical profile using techniques such as high-performance liquid chromatography mass spectrometry (HPLC-MS) or gas chromatography mass spectrometry (GC-MS) to correlate specific constituents with the observed antihyperglycemic and pancreatic tissue-preserving effects.

## 5 Conclusion

Oral *L. sativum* oil (250 mg/kg/day for 28 days) alleviates hyperglycemia, dyslipidemia, and serum oxidative stress markers while preserving pancreatic tissue architecture in streptozotocin-induced, type 1-like diabetic rat. It also improves selected serum markers of liver and kidney function (ALT, AST, urea, creatinine, and uric acid). However, the PDX-1 and GCK genes exhibited a non-significant change relative to untreated diabetic rats, suggesting that the observed benefits in this model may not be primarily mediated through transcriptional regulation of these genes. Collectively, *L. sativum* oil may play an antihyperglycemic and pancreatic tissue-preserving role in alleviating diabetes. Further research is needed to elucidate its bioactive constituents, molecular targets, and long-term safety.

## Data Availability

The original contributions presented in the study are included in the article/supplementary material. Further inquiries can be directed to the corresponding author.
